# Mixed allele malaria vaccines: Host protection and within-host selection

**DOI:** 10.1016/j.vaccine.2008.09.004

**Published:** 2008-11-11

**Authors:** Victoria C. Barclay, Brian H.K. Chan, Robin F. Anders, Andrew F. Read

**Affiliations:** a School of Biological Sciences, University of Edinburgh, Edinburgh EH9 3JT, UK; b Department of Biochemsitry, La Trobe University, Melbourne 3086, Australia; c Centre for Infectious Disease Dynamics, Departments of Biology and Entomology, The Pennsylvania State University, University Park 16802, USA

**Keywords:** Malaria, AMA-1, Strain-specific immunity, Virulence

## Abstract

Malaria parasites are frequently polymorphic at the antigenic targets of many candidate vaccines, presumably as a consequence of selection pressure from protective immune responses. Conventional wisdom is therefore that vaccines directed against a single variant could select for non-target variants, rendering the vaccine useless. Many people have argued that a solution is to develop vaccines containing the products of more than one variant of the target. However, we are unaware of any evidence that multi-allele vaccines better protect hosts against parasites or morbidity. Moreover, selection of antigen-variants is not the only evolution that could occur in response to vaccination. Increased virulence could also be favored if more aggressive strains are less well controlled by vaccine-induced immunity. Virulence and antigenic identity have been confounded in all studies so far, and so we do not know formally from any animal or human studies whether vaccine failure has been due to evasion of protective responses by variants at target epitopes, or whether vaccines are just less good at protecting against more aggressive strains.

Using the rodent malaria model *Plasmodium chabaudi* and recombinant apical membrane antigen-1 (AMA-1), we tested whether a bi-allelic vaccine afforded greater protection from parasite infection and morbidity than did vaccination with the component alleles alone. We also tested the effect of mono- and bi-allelic vaccination on within-host selection of mixed *P. chabaudi* infections, and whether parasite virulence mediates pathogen titres in immunized hosts. We found that vaccination with the bi-allelic AMA-1 formulation did not afford the host greater protection from parasite infection or morbidity than did mono-allelic AMA-1 immunization. Mono-allelic immunization increased the frequency of heterologous clones in mixed clone infections. There was no evidence that any type of immunization regime favored virulence. A single AMA-1 variant is a component of candidate malaria vaccines current in human trials; our results suggest that adding extra AMA-1 alleles to these vaccines would not confer clinical benefits, but that that mono-allelic vaccines could alter AMA-1 allele frequencies in natural populations.

## 1 Introduction

Malaria parasite antigens which are the targets of protective immune responses are frequently polymorphic, with antigen-coding genes having multiple allelic forms [1]. Polymorphisms likely arise as a consequence of immune-mediated selection because host responses can be more effective against parasites of the immunising strain than against different strains (strain-specific immunity) [2–6]. Sequence polymorphisms have been directly implicated in antigenic escape [7–9], and in malaria endemic areas, immunity is acquired slowly, probably because repeated exposure is required to generate an effective response against a repertoire of strains [10–12]. The existence of antigenic polymorphism is therefore of considerable concern to malaria vaccine developers because it implies that single antigen vaccines will have trouble inducing protective immunity against polymorphic targets [13–16].

One approach to minimizing vaccine-induced strain-specificity has been to design vaccines which combine more than one allele of an antigen [1,17–19]. However, the inclusion of more than one allelic form of an antigen may not be sufficient to overcome substantial polymorphisms [9], and there is little experimental evidence that multi-allele vaccines actually afford the host more protection from morbidity than do single antigen vaccines.

Furthermore, selection of antigen-variants is not the only evolution that could occur in response to widespread vaccination. Theoretically, vaccination has the potential to cause evolutionary change in parasite virulence (parasite-induced host damage) by altering the way natural selection acts on parasite populations [20–27]. In experimental evolution experiments, the rodent malaria *Plasmodium chabaudi* became virulent more rapidly if serially passaged through mice previously immunized with live parasites [28]. The most likely explanation for this is that more aggressive variants are less well controlled by immunity.

To date, we still do not fully understand how vaccines will alter gene frequencies in malaria parasite populations. Evidence for selection in the field comes from a small phase 1-2b trial of the “Combination B” blood-stage malaria vaccine [29]. This vaccine contained a single antigen from each of three polymorphic loci of *P. falciparum*. One of these loci, merozoite surface protein-2 (MSP-2), is dimorphic, with each parasite having an allele from one of two allelic families (labelled 3D7 and FC27). The MSP-2 allele in the Combination B vaccine came from the 3D7 family. Among parasites subsequently acquired by vaccines, 3D7-type alleles were rarer than in people given a placebo. Vaccination thus selected against the variant contained in the vaccine. Interestingly, the FC27 allelic family is associated with more virulent infections [30]. Therefore, it is not clear whether the vaccine-imposed selection was due to immune specificity [15,31] or whether the vaccine was less good at controlling more virulent infections.

Many candidate vaccines against malaria are directed against the asexual blood stage, with the principal target being the merozoite. Apical merozoite antigen-1 (AMA-1) is a promising vaccine candidate as it possesses fewer polymorphisms than other merozoite antigens [2,13]. AMA-1 is thought to play a major role during erythrocyte re-modelling and invasion [32]. Immunization with AMA-1 confers protection against parasite challenge in a number of animal models, probably by inducing antibodies which inhibit invasion [2,7,33–36]. Furthermore, humans and other species immunized with single allele AMA-1 vaccines raise antibodies which inhibit erythrocyte invasion in vitro [13,37]. In endemic populations, naturally acquired antibody to *P. falciparum* AMA-1 (PfAMA-1) is associated with protection from falciparum malaria [38–42]. At least six different vaccines based on the AMA-1 allele from the *P. falciparum* 3D7 strain are currently in efficacy trials in humans [43–45].

However, there are more than 60 polymorphic sites in the AMA-1 protein, and most of these are non-randomly dispersed point mutations on domain I [46–50]. These point mutations may be of immunological importance. Protection in mice is strain-specific, and growth and invasion inhibition assays (GIA) and ELISA show that antibodies from animals and human field sera inhibit growth in a strain-specific manner [2,13,34,38,43,47]. Allelic replacement experiments have directly implicated sequence polymorphism in antigenic escape [7], and cross-strain inhibition assays suggest that the extent of escape correlates with sequence distance between the vaccine and the target strain [8]. In an attempt to overcome strain-specificity, vaccine researchers are beginning to combine allelic variants of AMA-1. For example, one group immunized rhesus monkeys with a mixture of two allelic forms of PfAMA-1 (designated AMA-1-C1) or the component alleles and measured responses in vitro using GIA and ELISA [8,51,52]. The resulting antibodies were similarly effective regardless of whether immunization was with a single variant or AMA-1-C1. Another group immunized mice and rabbits with two allelic variants of domain I and II of AMA-1 ectodomain from *P. falciparum* isolates. The anti-AMA-1 antibodies obtained with both proteins were active in an in vitro parasite growth invasion/inhibition assay, but to no greater extent than with either of the variants alone [53]. Together these results have raised questions about the necessity of using multi-allele vaccines.

Here we use the rodent malaria *P. chabaudi* and two alleles of the blood-stage malaria vaccine candidate AMA-1 to investigate (i) whether immunization with a single or bi-allelic AMA-1 variant formulation afforded the host the greatest protection from morbidity and parasite infection, (ii) how these different vaccination regimes can alter clonal frequencies in mixed infections, and (iii) whether more virulent clones are better at evading heterologous vaccine-induced protective responses.

## 2 Materials and methods

### 2.1 Parasites and hosts

*P. chabaudi adami* clones were originally derived from wild-caught thicket rats (*Thamnomys rutilans*) in the Congo and stored as frozen stabilites in liquid nitrogen with subscript codes used to identify their position in clonal history [54,55]. In this experiment we used clones DS_500_ and DK_122_ originally cloned from isolates 408XZ and 556KA, respectively. The nucleotide sequences of the DK and DS *P. chabaudi* AMA-1 (PcAMA-1) gene differ at 79 sites [34,56]. Hosts were inbred female C57BL/6 mice aged 6–8 weeks (Harlan, England) maintained as described previously [57]. Studies by others [34] and our own pilot studies showed that these clones differ in virulence during the infection of C57BL/6 female mice, with clone DS generating substantially more parasites and inducing greater weight and red blood cell loss relative to DK.

### 2.2 Immunizations and isotype ELISA

Here we used an immunization protocol adapted from Anders et al. [2]. Prior to immunization, mice were randomized into four groups of eighteen (Table 1). Immunization was with the highly immunogenic ectodomian of the full AMA-1 protein termed AMA-1B. For mono-allelic immunizations (hereafter referred to as DS AMA-1 or DK AMA-1), groups of mice were injected intraperitoneally with 10 μg of the appropriate protein emulsified in 100 μl of the adjuvant Montanide ISA720 (Seppic, France). For bi-allelic immunizations, mice were injected with a mixture of 5 μg of both DS and DK AMA-1, giving the same total dose of antigen as for the single antigen immunizations, again emulsified in Montanide ISA720. Control mice were injected with 100 μl emulsion of PBS in Montanide ISA720. Mice were given a single booster immunization with the same amount of antigen emulsified in Montanide ISA720 4 weeks after the primary immunization.

To ensure that antigen immunization successfully generated antibody responses, and to determine whether there was any cross-reactivity between the antibodies generated to the different immunizing antigens, we first carried out a pilot experiment. A total of 11 mice were immunized with DS AMA-1, 11 with DK AMA-1, and 10 were sham-immunized. We estimated the quantity of IgG2b antigen-specific antibodies in all mice sera 11 days after the booster immunization by ELISA using wells coated with DS AMA-1 or DK AMA-1. Thus the sera from 32 mice were tested in 64 wells. We used IgG2b as previous work in our laboratory showed that C57BL/6 produce this isotype in response to *P. chabaudi* infection (K. Grocock, A. Graham, unpublished). Protection induced by immunization with recombinant AMA-1 is isotype independent [58b]. Given the lack of cross-reactivity we observed in this pilot experiment (see Section 3), in the main experiment, we measured IgG2b isotype antibodies to each antigen separately only from the sera of mice immunized with a mixture of DS and DK AMA-1 and in sham-immunized control mice.

In both the pilot and main experiments, sera fractions were separated by centrifugation from 20 μl of blood taken from a tail snip and were stored at −80 °C. High binding 96 well ELISA Maxisorb immunoplates (Nunc) were coated with either DS AMA-1 or DK AMA-1 at a concentration of 1 μg/ml in 0.06 M carbonate buffer (0.04 M NaHCO_3_, 0.02 M NaCO_3_, pH 9.6) in a final volume of 50 μl per well. Plates were stored at 4 °C overnight to allow the antigen to bind. Non-specific binding was blocked by incubating wells with 5% BSA: carbonate buffer (200 μl per well) for 2 h at 37 °C. Wells were then washed three times in Tris buffered saline with 0.01% Tween 20 (TBST). We used end-point dilution methods to detect IgG2b titres: serum samples were detected in a serial dilution 1/100-1/204800 using TBST as a diluent, in a final volume of 50 μl per well and incubated for 2 h at 37 °C. Wells were washed three times in TBST. HRP conjugated goat anti-mouse IgG2b detection antibody (Southern Biotech 1100-05) was diluted 1/4000 in TBST to a final volume of 50 μl per well. Plates were incubated for 1 h at 37 °C. Wells were washed three times in TBST followed by a final wash in distilled water. ABTS peroxide substrate (Insight Biotechnology) was added at 100 μl per well and allowed to develop at room temperature for 20 min. Optical density was read at 405 nm using a spectrophotometer. IgG2b isotype antibody titres were calculated as the reciprocal of the greatest dilution at which optical density (O.D.) was greater than the mean (plus 2 standard deviations) O.D. values observed for naïve mouse sera assayed against both DS and DK AMA-1 at 1/100.

### 2.3 Parasite challenge and monitoring of within-host dynamics

Two weeks after the boost immunization, groups of immunized mice (18 per group) were further randomized into groups of six and challenged with 10^5^ parasites of either clone DS alone, clone DK alone or a mixture of clone DS and DK (Table 1). Thus, mice infected with both clones received twice as many parasites as those infected with one clone. A two-fold difference in infective dose has negligible effects on the population dynamics of the parasite [58a]. During the course of infection, we measured body weights and took blood samples from the tail to (i) make Giemsa-stained blood smears, (ii) estimate red blood cell density by flow cytometry (Beckman Coulter), and (iii) for genotype-specific real-time quantitative PCR (qPCR) assays as described previously [59]. For amplification of the DK genotype, we used primers previously designed to amplify AS/AJ genotypes as described elsewhere [59]. DS genotype-specific primers were as follows: DS forward 5′-GGA AAA GGT ATA ACT AAT CAA AAA TCT ACT AAA-3′; DS reverse 5′-CAG GAG AAA TGT TTA CAT CTG CTT T-3′.

### 2.4 Trait definition and statistical analyses

Since *P. chabaudi* has a 24-h replication cycle, the total number of parasites present in any period can be estimated by summing the daily parasite counts. Data were analysed using General Linear Models (GLMs) in MINITAB. To meet normality and homogeneity of variance assumptions, data on antibodies, weight and red blood cell density were log transformed while all parasite densities and proportions were square root transformed. GLMs were used to test whether the magnitude of protection differed between the three antigen immunizations (DK AMA-1, DS AMA-1, or the bi-allelic form); that is whether there was a statistical interaction between infecting clone and immunizing treatments. Maximal models (response variable = infecting clone + immunizing treatment + infecting clone × immunization treatment) were tested in the first instance, and minimal models were obtained by dropping non-significant terms successively, beginning with highest order interactions, to obtain the significant minimal model. For analyses of within-host selection, we asked for mixed clone infections, whether the frequency of clone DS in the parasite population differed between the sham-immunized controls and the antigen immunizations.

## 3 Results

Table 1 gives details of the immunization treatments, infecting clone and sample size of the experiment. Some mice died; these were included in the calculation of daily densities until death, and in the analyses of peak parasite densities since death always occurred as initial parasiteamias were declining.

### 3.1 Pre-challenge anti-AMA-1 IgG2b antibodies

**Fig. 1:**
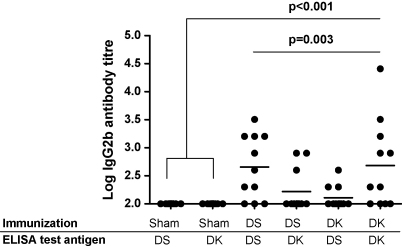
IGg2b antibody levels from the serum of mice in the pilot experiment. Mice were either sham-immunized or immunised with one of the two atingens (DS AMA-1, DK AMA-1). Each of the treatments used to immunize mice and the AMA-1 test antigen used to coat ELISA plates are shown on the *x*-axis. Dots represent the antibody titre against a particular immunizing antigen for a single mouse. Horizontal lines indicate mean antibody levels. Antibody levels in antigen immunized groups of mice were higher than in sham-immunized controls (*p* < 0.001) and, among the immunized mice, the levels induced between the antigen immunized groups differed (immunizing treatment × ELISA antigen: *p* = 0.003) with higher titres against the homologous antigen. Neither of the immunising antigens induced higher titres (*p* > 0.05).

Fig. 1 illustrates the data from a pilot experiment where IgG2b antigen-specific antibodies were measured to each of the immunizing antigens and the cross-reactivity between them. All antigen immunization treatments generated antibody titres that were higher than those present in sham-immunized controls (sham-immunized versus antigen immunized: *F*_1,62_ = 8.92, *p* = 0.004). IgG2b antibodies were specific for the antigen they had been exposed to during immunization (immunizing treatment × ELISA antigen: *F*_1,40_ = 9.99, *p* = 0.003). For example, anti-DS AMA-1 IgG2b antibody tires were higher when assayed against the homologous DS antigen than the heterologous DK antigen and vice versa. Thus, neither antigen elicited a stronger response overall.

**Fig. 2:**
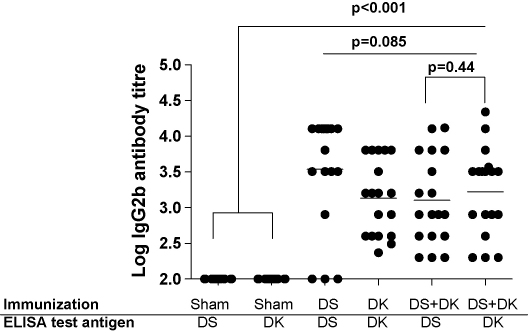
IgG2b antibody levels from the serum of mice in the main experiment. Mice were either sham-immunized, or immunized with one of the antigen immunization treatments (DS AMA-1, DK AMA-1 or the bi-allelic formulation). Each of the treatments used to immunize mice and the AMA-1 test antigen used to coat ELISA plates are shown on the *x*-axis. Dots represent the antibody titre for individual mice against a particular antigen. Mice that were sham-immunized or immunized with the bi-allelic formulation were assayed for antibody responses against both DS and DK AMA-1 antigens. Horizontal lines indicate mean antibody levels. Antibody levels in antigen immunized groups of mice were higher than in sham-immunized controls (*p* < 0.001), and among the antigen immunized mice, antibody titres did not differ (*p* = 0.085). The antibody titres in animals immunized with both antigens were not dominated by responses to either one (*p* = 0.44).

Fig. 2 illustrates the IgG2b antibody titres in mice from the main experiment 3 days prior to parasite infection. All antigen immunization treatments induced antibody titres that were higher than those present in sham-immunized mice (sham-immunized versus immunized: *F*_1,106_ = 58.89, *p* < 0.001). Among antigen-immunized groups, titres did not differ (*F*_2,69_ = 2.56, *p* = 0.085). In those mice which had been immunized with the bi-allelic form, antibodies were not more specifically recognising either component antigen (*F*_1,35_ = 0.61, *p* = 0.44). These data show that immunization successfully elicited antibody responses, and that, at least as measured by IgG2b titres, these responses were of equal magnitude in all immunized groups. Among antigen-immunized mice, antibody titres prior to challenge did not predict subsequent parasite intensities, weight loss or anaemia (all correlations, *p* > 0.2).

### 3.2 Bi-allelic immunization did not generate a greater anti-morbidity response than did mono-allelic immunization

**Fig. 3:**
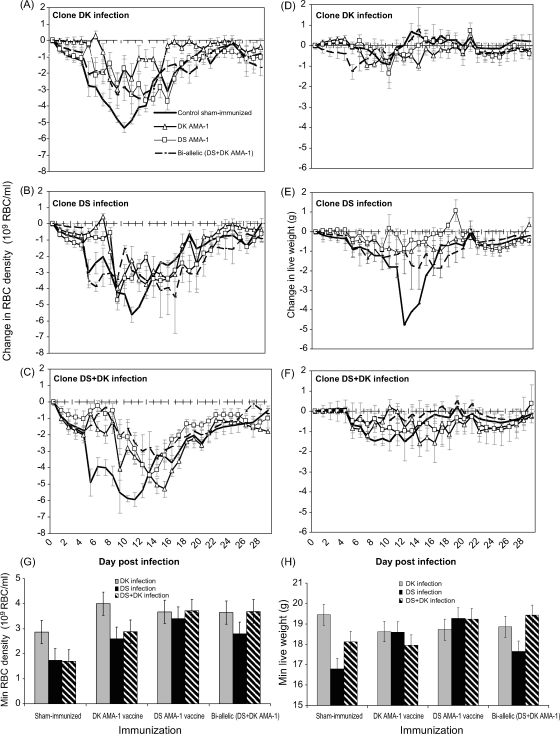
Effect of *Plasmodium chabaudi* infection (clone DK alone, DS alone or DS + DK) and immunization (sham-immunized control, DK AMA-1, DS AMA-1, or bi-allelic form) on the kinetics of minimum red blood cell density (left panels) and minimum weight (right panels). In A–F lines represent the change in RBC density (left panels) and weight (right panels) over time. Each line represents the mean of up to 6 mice (±1 S.E.M.) that were infected with DK alone (A and D), DS alone (B and E) or a mixed clone (C and F) infection during immunization with either a sham-inoculation control (solid thick black line), DK AMA-1 (open triangle), DS AMA-1 (open squares), or the bi-allelic mixture (dotted black line). In G–H bars represent the minimum red blood cell density (left panel) and minimum weight (right panel) reached during infection with clone DK alone (grey bars), DS alone (black bars) or a mixture of both clones (black and white bars) under each of the immunization treatments. Each bar represents the least squares mean of up to 6 mice (±1 S.E.M.).

Red blood cell density and weight kinetics following parasite challenge for each of the immunization treatments are illustrated in Fig. 3A–F, and the minimum red blood cell density and minimum weight reached are illustrated in Fig. 3G–H. In sham-immunized control mice, clone DK was less virulent than clone DS, induced less anaemia and less weight loss (Fig. 3A–H; anaemia: *F*_2,14_ = 6.29, *p* = 0.011; weight loss: *F*_2,14_ = 9.97, *p* = 0.002).

Immunization protected mice against anaemia induced by infection with any of the clones (Fig. 3A–C, G; sham-immunized versus immunized: *F*_1,69_ = 16.94, *p* < 0.001). Bi-allelic immunization reduced anaemia no more than did immunization with either of the alleles alone (Fig. 3G; immunizing treatment × infecting clone: *F*_4,44_ = 0.71, *p* = 0.59). All pairwise immunization comparisons were non-significant (*p* > 0.5 in all cases).

As infection with clone DK did not induce any weight loss in sham-immunized controls (Fig. 3D) the protective effects of immunization were analysed only for infections that contained clone DS (Fig. 3E–F). We found that all immunizations protected mice against weight loss due to DS infections (Fig. 3E–F, H; sham-immunized versus immunized: *F*_1,45_ = 11.13, *p* = 0.002). Similar to the anaemia data, we found that immunization with either the bi-allelic form or either of the alleles alone afforded similar levels of protection against weight loss (Fig. 3H; immunizing treatment × infecting clone: *F*_2,29_ = 2.43, *p* = 0.11). All pairwise immunization comparisons were non-significant (*p* > 0.5 in all cases).

Together, these results show that immunization with the bi-allelic vaccine does not afford the host greater protection from morbidity, as measured by anaemia and weight loss. Immunization with either of the variants alone provided protection which was as effective as that induced by the two variants together.

### 3.3 Bi-allelic immunization did not generate greater anti-parasite response than did mono-allelic immunization

**Fig. 4:**
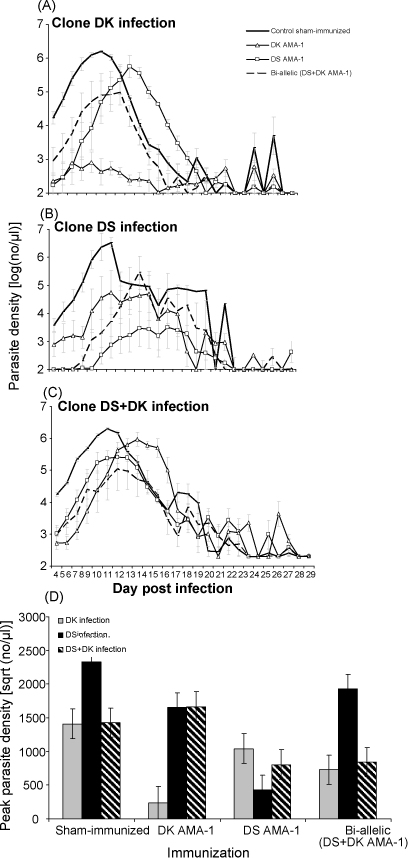
Kinetics of *P. chabaudi* infections (clones DK alone, DS alone or both together) following immunization (DK AMA-1, DS AMA-1, or bi-allelic formulation or sham-immunized control). In A–C, lines represent the change in parasite density over time. Each line represents the mean of up to 6 mice (±1 S.E.M.) that were infected with DK alone (A), DS alone (B) or a mixed clone (C) infection during immunization with either a sham-inoculation control (solid thick black line), DK AMA-1 (open triangle), DS AMA-1 (open squares), or the bi-allelic mixture (dotted black line). In (D), bars represent peak parasite densities reached during infection with clone DK alone (grey bars), DS alone (black bars) or a mixture of both clones (black and white diagonal) under each of the immunization treatments. Each bar represents the least squares mean of up to 6 mice (±1 S.E.M.).

Parasite dynamics under each of the treatments are illustrated in Fig. 4. Clone DS achieved higher parasite density in sham-immunized control mice than did clone DK (infecting clone: *F*_1,10_ = 7.03 = 0.024).

All three immunizations reduced peak parasite densities relative to those which had received a sham inoculation (Fig. 4D; sham-immunized versus immunized: *F*_1,69_ = 11.55, *p* = 0.001). The extent of anti-parasite protection depended on the identity of the immunising antigen and the identity of the challenge clone (Fig. 4D; immunizing treatment × infecting clone: *F*_4,44_ = 8.71, *p* < 0.001). We found that protection was clone-specific: immunization with DS AMA-1 antigen reduced DS parasite densities more than it reduced the densities of clone DK, and vice versa (among single antigen immunized groups, immunizing treatment × infecting clone: *F*_1,19_ = 36.26, *p* < 0.001).

When we compared the extent of anti-parasite protection between the immunized groups we found that under no circumstances did the bi-allelic immunization afford greater protection than did immunization with a single allele. For example, immunization with DS AMA-1 reduced the peak density of DK infections and infections with both clones together, but the bi-allelic immunization did not protect against DS alone (Fig. 4D; immunizing treatment × infecting clone: *F*_2,30_ = 9.84, *p* = 0.001). Although the bi-allelic immunization reduced the densities of clone DK, reduction was no greater than with a single DK AMA-1 immunization (Fig. 4D; immunizing treatment × infecting clone *F*_2,29_ = 4.09, *p* = 0.027).

Together these results show that bi-allelic immunization did not afford the host greater anti-parasite protection than did mono-allelic immunization. Unlike morbidity, where protection was induced regardless of the antigen used in immunization, we found that immunization with a single allele achieved better protection against the homologous clone, and bi-allelic immunization never did as well. Indeed, we found just one of the variants (DS AMA-1) to be the most effective at reducing parasite densities.

### 3.4 Vaccine-induced anti-parasite protection was clone specific in mixed infections and independent of clone virulence

**Fig. 5:**
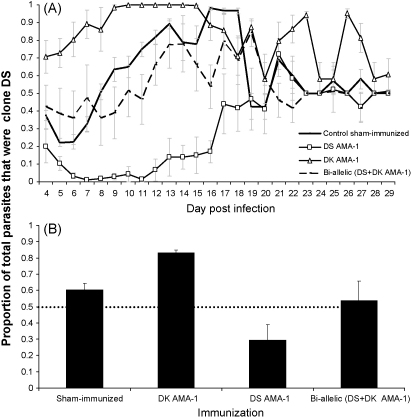
Proportion of clone DS in mixed DS and DK infections following immunization with DK AMA-1, DS AMA-1, the bi-allelic formulation, or in sham-immunized controls. (A) Lines represents the proportion of clone DS through time in control (solid black line), DK AMA-1 (open triangles), DS AMA-1 (open diamonds) or bi-allelic (dotted black line) immunized mice. Each line represents the mean of up to 6 mice (±1 S.E.M.). (B) Bar graphs represent the proportion of total parasites in a mixed infection that were DS under each of the immunization treatments. Each bar represents the least squares mean of up to 6 mice with 95% confidence intervals. The black horizontal dotted line represents the proportion DS present in the inoculum.

To examine how the antigenic composition of the immunizing formulation affects within-host selection (relative frequency) in mixed clone infections, and whether heterologous immunity less effectively controlled the virulent clone, we compared the frequency of clone DS in mixed infections (Fig. 5).

We found that antigen immunization altered clone frequencies. In sham-immunized mice, and those immunized with the bi-allelic formulation, DS made up about 60% of all the parasites present in the infections. Thus, immunization with a mixture of DS and DK AMA-1 had negligible effect on clone frequency and thus within-host selection (Fig. 5A; sham-immunized versus bi-allelic immunization: *F*_1,10_ = 2.02, *p* = 0.19). In contrast, immunization with a single antigen reduced parasites in a clone-specific manner, facilitating the heterologous clone (Fig. 5A and B; immunizing treatment *F*_1,10_ = 105.54, *p* < 0.001). Immunization with DK AMA-1 increased the frequency of clone DS, while DS AMA-1 immunization increased the frequency of DK. These effects were essentially symmetrical. Thus, there was no evidence that the more virulent clone, DS, was less affected by heterologous immunization than was the less virulent clone DK.

## 4 Discussion

In this study, we investigated (i) whether immunization with a single or bi-allelic AMA-1 formulation afforded the host the greatest protection from morbidity and parasite infection, (ii) how these different vaccination regimes altered clonal compositions in mixed infections, and (iii) whether a more virulent clone was less successfully controlled by vaccine-induced protective responses. Addressing each of these in turn, we found the following. (i) Bi-allelic immunization did not generate better anti-morbidity or anti-parasite protection than did single allele immunization. Rather, immunization with one of the two variants alone (DS) provided the best protection. (ii) Both single variant immunizations reduced the frequency of homologous clones in mixed infections; bi-allelic immunization had no impact on within-host selection. (iii) There was no evidence that the more virulent clone (DS) was better at evading vaccine-induced immunity than was the less virulent clone.

Rightly, protecting individual hosts from morbidity is one of the goals of malaria vaccines directed against the blood-stage of infection. If infection densities are positively correlated with host morbidity (virulence) [27] multi-allele vaccines could potentially improve the health of the host by suppressing more of the parasite population and reducing strain-specific responses. Subject to the usual cautions about generalising from animal models (reviewed in this context by Råberg et al. and Wargo et al. [60,61]), the results presented here argue against that, and suggest that protective efficacy may not be increased by including alternative variants of AMA-1. Our in vivo observations are consistent with previous results showing that immunization of rhesus monkeys with only one of two PfAMA-1 variants is sufficient to induce cross-protective antibody responses as measured by GIA and ELISA assays in vitro [52]. Our results are also consistent with another study which demonstrated that mice and rabbits immunized with two allelic variants of domain I and II of the full length AMA-1 ectodomain from Indian *P. falciparum* isolates were able to inhibit in vitro parasite growth, but to no greater extent than with either of the allelic variants alone [53].

Our results also demonstrate strain-specific anti-parasite responses (Fig. 4D) need not result in strain-specific protection against disease (Fig. 3G and H). The observation that there are two different types of anti-malarial responses – immunity against the parasite itself and immunity against disease – is poorly understood on a molecular basis although the distinction is widely appreciated [62]. An explanation for the two different responses observed here could be that the specificity of the anti-AMA-1 antibody response lies with the generation of inhibitory antibodies which may target the hypervariable region located around a conserved hydrophobic pocket on domain I [63]. The presence of such antibodies could determine the observed parasitaemias. For bi-allelic immunizations there may exist a dominant epitope in one allelic form of AMA-1. Thus, high titres of cross-reactive antibodies may be sufficient to lessen morbidity (hence the similar effects for mono-and-bi-allelic vaccination on morbidity) but the inhibitory antibodies are more effective at controlling parasite numbers by inhibiting invasion. In our pilot studies we did not observe a disproportional IgG2b antibody response to one of the immunizing antigens (Fig. 1). However, since immunization with AMA-1 is likely to induce a repertoire of IgG isotypes [58a,64–66] some of the other isotypes may be sufficiently cross-reactive. An implication of this may be that while strain-specific immunization may alter allele frequencies in parasite populations, this need not have clinical consequences in a vaccinated host. Changes in allele frequencies without public health consequences have been seen in some other diseases, such as pertussis (reviewed in [26]).

The ‘Combination B’ malaria vaccine, one of the few to reach field trials, demonstrated strain-specific anti-parasite effects despite being comprised of an allele of each of 3 asexual blood stage proteins, MSP-1, MSP-2 and RESA (ring-infected erythrocyte surface antigen) [29]. Of particular interest was that vaccination increased the frequency of parasites with an MSP-2 genotype belonging to the FC27 allelic family. No representatives of this allelic family, which had been found previously to be associated with severe morbidity, were included in the vaccine [16,29,30]. Selection for the FC27 form of MSP-2 could have been because of strain-specific protection [15,31], or because the vaccine was less effective at protecting against more virulent strains [26–28]. In the study we report here, we looked at the relative proportion of the more virulent clone in a mixed infection under the different immunization compositions. In sham-immunized control mice and those which received the bi-allelic immunization, the more virulent clone (DS) was proportionally the most dominant. Thus, bi-allelic immunization did not alter within-host selection. On the other hand, immunization with a single AMA-1 variant did facilitate evasion of the heterologous clone in mixed infections. In our experiments, this effect was symmetrical (Fig. 5), so that immunization with AMA-1 appears to induce protective responses that are strain-specific and evasion is independent of parasite virulence.

Nevertheless, selection for virulence could be an inadvertent consequence of including just one allele from a given locus in a vaccine, as apparently happened in the Combination B trial. As far as we are aware, there are no reports that variants of AMA-1 have different intrinsic virulence, so that the strain-specific immunity against this locus we report here and that has been seen by others [7,34], should not directly alter virulence. But caution is necessary for all antigens involved in processes like cell invasion which are associated with pathogenesis. Population-level association studies for disease severity should be performed for all antigens included in candidate vaccines. Should associations like that for MSP-2 be found [30], we suggest on the basis of our results that there would be a strong case for including all known variants at that locus in the vaccine. This would not confer short-term clinical advantage, but it would be the safest way to avoid inadvertent selection for virulent variants, which would put unvaccinated people at greater risk.

More generally, though, we still have some way to go to understand the potential for vaccine-driven virulence evolution, even in the *P. chabaudi* model. One experimental study demonstrated that parasites from a single *P. chabaudi* clone serially passaged through whole-parasite immunized mice evolved to be more virulent than those evolved in naive hosts [28]. That study was the first to show under controlled conditions that immunization can favour the evolution of more virulent parasites. The implication was that more virulent variants had a selective advantage in immunized hosts. In the study we report here, which did not involve serial passage, we saw no signs of such an advantage. DS, the more virulent clone, dominated in mice immunized with the bi-allelic form, but to the same extent as in non-immunized mice. In single antigen immunized mice, strain-specific immunity dominated with symmetrical effects for both clones. Competition experiments with other *P. chabaudi* clones also failed to find an increased advantage to virulence in immunized hosts [67]. It may be that the accelerated evolution of virulence seen during serial passage in immunized hosts [28,68] is a feature of selection of virulence variants on an antigenically identical background. In future experiments, we will serially passage single *P. chabaudi* clones through AMA-1 immunized and naïve mice to determine whether vaccination can evolve virulence to be greater when measured in naïve hosts.

Our experiments concerned antigenic polymorphism at a single target antigen. Considerably more work has focused on vaccines combining single variants from multiple antigenic loci [1,69,70]. For example, animal and human phase I trails have shown safety, tolerability and immunogenecity of formulations containing AMA-1 and MSP-1 [1,71–73]. Moreover, such ‘multi-valent’ vaccines have been shown to reduce parasitaemias in mice of distinct MHC haplotypes [74] and against infections with different parasite strains as well as subspecies of different virulence [75]. Thus, multi-valency may be required to induce antibody responses against a repertoire of polymorphic parasite antigens [64,66,76–81] in the human outbred population exposed to multiple parasite genotypes [29,78,82–84]. We suspect that multi-valent vaccines will prove to be a more efficient means of generating protection against the widest range of parasite genotypes. Certainly, we found no evidence that the anti-morbidity and anti-parasitic potency of a malaria vaccine would be enhanced by increasing the number of variants of a particular antigen.

## Figures and Tables

**Table 1:** Experimental design

	Number of mice per immunization	Infecting clone	Number of mice per parasite infection	Number of deaths	Number of euthanized
Sham-immun	18	DS	6	3	2
Sham-immun		DK	6		
Sham-immun		DS + DK	6		
DK AMA-1	18	DS	6		
DK AMA-1		DK	6		
DK AMA-1		DS + DK	6		
DS AMA-1	18	DS	6	1	
DS AMA-1		DK	6		
DSAMA-1		DS + DK	6	1	
Bi-allelic	18	DS	6	2	1
Bi-allelic		DK	6		
Bi-allelic		DS + DK	6		
Total	72		72	7	3

Immunization was either with DK AMA-1, DS AMA-1, a formulation containing an equal mix of both forms of AMA-1 (bi-allelic), or immunization with adjuvant only (‘sham-immunization’). Groups of 18 mice were immunized with one of the four treatments before being separated into groups of 6. Infection was with parasites of clone DK alone, clone DS alone or a mixture of both. During the experiment 7 mice were found dead and 3 had to been euthanized due to severe morbidity. Euthanization was at predetermined levels of morbidity prescribed by animal care protocols.
